# Evidence-based clinical practice guidelines for peptic ulcer disease 2020

**DOI:** 10.1007/s00535-021-01769-0

**Published:** 2021-02-23

**Authors:** Tomoari Kamada, Kiichi Satoh, Toshiyuki Itoh, Masanori Ito, Junichi Iwamoto, Tadayoshi Okimoto, Takeshi Kanno, Mitsushige Sugimoto, Toshimi Chiba, Sachiyo Nomura, Mitsuyo Mieda, Hideyuki Hiraishi, Junji Yoshino, Atsushi Takagi, Sumio Watanabe, Kazuhiko Koike

**Affiliations:** 1 Department of Health Care Medicine, Kawasaki Medical School General Medical Center, 2-6-1, Nakasange, Kita-ku, Okayama, 700-8505 Japan; 2 Guidelines Committee for Creating and Evaluating the ‘‘Evidence-Based Clinical Practice Guidelines for Peptic Ulcer,” the Japanese Society of Gastroenterology (JSGE), 6F Shimbashi i-MARK Bldg., 2-6-2 Shimbashi, Minato-ku, Tokyo, 105-0004 Japan

**Keywords:** Peptic ulcer, *Helicobacter pylori* eradication, Nonsteroidal anti-inflammatory drug, Low-dose aspirin, Idiopathic ulcer

## Abstract

The Japanese Society of Gastroenterology (JSGE) revised the third edition of evidence-based clinical practice guidelines for peptic ulcer disease in 2020 and created an English version. The revised guidelines consist of nine items: epidemiology, hemorrhagic gastric and duodenal ulcers, *Helicobacter pylori* (*H. pylori*) eradication therapy, non-eradication therapy, drug-induced ulcers, non*-H. pylori,* and nonsteroidal anti-inflammatory drug (NSAID) ulcers, remnant gastric ulcers, surgical treatment, and conservative therapy for perforation and stenosis. Therapeutic algorithms for the treatment of peptic ulcers differ based on ulcer complications. In patients with NSAID-induced ulcers, NSAIDs are discontinued and anti-ulcer therapy is administered. If NSAIDs cannot be discontinued, the ulcer is treated with proton pump inhibitors (PPIs). Vonoprazan (VPZ) with antibiotics is recommended as the first-line treatment for *H. pylori* eradication, and PPIs or VPZ with antibiotics is recommended as a second-line therapy. Patients who do not use NSAIDs and are *H. pylori* negative are considered to have idiopathic peptic ulcers. Algorithms for the prevention of NSAID- and low-dose aspirin (LDA)-related ulcers are presented in this guideline. These algorithms differ based on the concomitant use of LDA or NSAIDs and ulcer history or hemorrhagic ulcer history. In patients with a history of ulcers receiving NSAID therapy, PPIs with or without celecoxib are recommended and the administration of VPZ is suggested for the prevention of ulcer recurrence. In patients with a history of ulcers receiving LDA therapy, PPIs or VPZ are recommended and the administration of a histamine 2-receptor antagonist is suggested for the prevention of ulcer recurrence.

## Introduction

In 2009, the Japanese Society of Gastroenterology (JSGE) developed evidence-based clinical practice guidelines for peptic ulcer disease. The guidelines were revised in 2015 and again in 2020. Of the 90 clinical questions (CQs) included in the previous guidelines, those with a clear conclusion were considered background questions (BQs) and those requiring future research were considered future research questions (FRQs) in this revised guideline. Thus, the revised guidelines consist of nine items (28 CQs and one FRQ), including, for the first time, epidemiology and remnant gastric ulcer. Both epidemiology and conservative therapy for perforation and stenosis included only BQ. The prevention of hemorrhagic peptic ulcers in patients taking antithrombotic drugs and the treatment of ischemic duodenal ulcers have been added as a CQ and FRQ, respectively.

A literature search of Medline and the Cochrane Library was performed for data regarding the CQs published between 1983 and October 2018, and the Igaku Chuo Zasshi databases were searched for data published between 1983 and December 2018. The guidelines were developed using the Grading of Recommendations Assessment, Development and Evaluation (GRADE) system [1]. The quality of evidence was graded as A (high), B (moderate), C (low), and D (very low). Recommendation strength was indicated as either a ‘‘strong recommendation’’ or a ‘‘weak recommendation’’ The systematic review (SR) team conducted meta-analysis (MA) and decided the total strength of the evidence from A to D. The consensus was previously defined as 70% or more votes in agreement.

In Japan, incidence of cerebral infarction and myocardial infarction are increasing, and many patients undergo antithrombotic therapy including dual antiplatelet therapy (DAPT). In endoscopic clinical practice, focus has shifted from the risk of gastrointestinal (GI) bleeding to thromboembolism associated with the withdrawal of antithrombotic therapy [2, 3]. In addition, for nearly 50 years, the only oral anticoagulants were vitamin K antagonists (warfarin); however, four non-vitamin K antagonist direct oral anticoagulants (DOACs) are currently available. The hemorrhagic gastric and duodenal ulcer section of the revised guidelines emphasizes methods for the discontinuation of antithrombotic therapy including DOACs and for the prevention of hemorrhagic ulcers in patients taking anticoagulant and antiplatelet drugs, such as DAPT.

Vonoprazan (VPZ) provides potent and long-lasting inhibition of gastric acid secretion, and its efficacy is, therefore, expected to be superior to that of proton pump inhibitors (PPIs). Recent reports have shown that triple therapy including VPZ is as effective as first-line and second-line therapies for the eradication of *Helicobacter pylori* [4]. Several reports have indicated that VPZ effectively heals peptic ulcers [5] and prevents the recurrence of nonsteroidal anti-inflammatory drugs (NSAIDs) [6] and low-dose aspirin (LDA)-related ulcers [7]. This revised version also emphasizes the clinical results of VPZ.

## Hemorrhagic gastric and duodenal ulcers

### Non-endoscopic hemostatic therapy

#### CQ-1

Treatment of with patients with hemorrhagic peptic ulcers if they prescribed anticoagulants and/or antiplatelet agents?

- Recommended that aspirin be continued for conditions at high risk for thromboembolic events.
  Recommendation: strong, 100% agreed, evidence level B.
- Suggested to change antiplatelet agents to aspirin in patients with conditions with a high risk of thromboembolic events.
  Recommendation: weak, 100% agreed, evidence level D.
- Suggested to suspend antiplatelet agents, except for in patients at high risk for thromboembolic events.
  Recommendation: weak, 100% agreed, evidence level D.
- Recommended to suspend warfarin, if necessary, in endoscopic hemostasis patients. If warfarin is discontinued, we suggest heparin or resuming warfarin as soon as hemostasis is established.
  Recommendation: strong, 100% agreed, evidence level C.
- Suggested to resume DOACs early (within 1–2 days) after confirming endoscopic hemostasis.
  Recommendation: weak, 100% agreed, evidence level D.
- In patients receiving both antiplatelet agents and warfarin, suggested to change antiplatelet agents to aspirin or cilostazol. Continue warfarin under a suitable prothrombin time-international normalized ratio (PT-INR) or to change warfarin to heparin.
  Recommendation: weak, 100% agreed, evidence level D.
- In patients receiving dual antiplatelet agents, recommended that aspirin alone should be continued.
  Recommendation: strong, 100% agreed, evidence level D.
  Comment: The risk of re-bleeding due to continuing anticoagulant and/or antiplatelet agents and the risk of thromboembolism associated with their withdrawal should be considered. One randomized controlled trial (RCT) [8] reported significantly lower mortality in the continuing LDA group compared with the group that did not. However, clinical evidence is currently lacking to support the management of patients who receive antiplatelet agents excluding aspirin, warfarin, and DOACs with peptic ulcer bleeding (PUB). We considered that these recommendations are based on expert opinions of the guidelines of Japan Gastroenterological Endoscopy, Asia–Pacific working group and European Society of Gastroenterological Endoscopy. It is necessary to collaborate closely with gastroenterologists and cardiologists as patients at high risk of thromboembolism with PUB could be unstable.

#### CQ-2

Is interventional radiology (IVR) effective in patients undergoing refractory endoscopic treatment for hemorrhagic peptic ulcers?

- In patients undergoing refractory endoscopic treatment for hemorrhagic peptic ulcers, interventional radiology (IVR) is suggested due to its safety and effectiveness.
  Recommendation: weak, 100% agreed, evidence level C.
  Comment: The effectiveness of IVR with transcatheter arterial embolization was proven by two MAs [9, 10]. They reported that compared to surgery, IVR exhibits a higher re-bleeding rate, but no significant difference in mortality, need for additional interventions, or complication rates between treatments. IVR could be a viable option for the treatment of refractory PUB; however, a limited number of institutions can perform IVR.

#### CQ-3

Is medication with antacid agents required after endoscopic treatment for hemorrhagic peptic ulcers?

- PPI administration after endoscopic treatment for hemorrhagic peptic ulcers is recommended to improve treatment outcomes.
  Recommendation: strong, 100% agreed, evidence level A.
  Comment: Compared with placebo, intravenous PPI therapy after endoscopic treatment for hemorrhagic peptic ulcers has been proven to reduce the rate of re-bleeding, volume of blood transfusion, period of admission, and rate of conversion to surgery in two MAs [11, 12] and some randomized controlled trials. The efficacy of high-dose PPI therapy is not higher than non-high-dose PPI regarding decreasing re-bleeding, surgical intervention, or mortality after post-endoscopic hemostasis [13].
  There was no significant difference effect between oral and intravenous PPI therapy on mortality, re-bleeding, need for blood transfusion, length of hospital stays, or surgery [14].
  Compared to intravenous histamine 2-receptor antagonist (H_2_RA) therapy, PPI therapy reduces rates of ulcer re-bleeding, surgical intervention, and overall duration of hospital stay with no significant difference in mortality [15]. Considering patient benefits, we recommended PPI therapy after endoscopic treatment for hemorrhagic peptic ulcers.

### Prevention of hemorrhagic peptic ulcer

#### CQ-4

What drugs are recommended for the prevention of hemorrhagic ulcers in antithrombotic users?

**Fig. 1:**
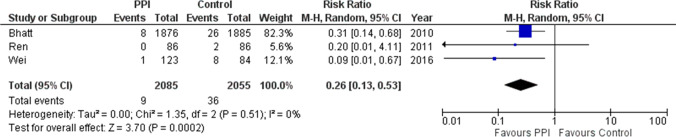
Forest plots of proton pump inhibitors (PPIs) against control for upper gastrointestinal bleeding preventive effect in dual antiplatelet therapy (DAPT). In meta-analysis, PPIs significantly have reduced bleeding risk compared to controls (RR 0.26; 95%CI 0.13–0.53, *P* = 0.0002)
  Only observational studies were available regarding the UGIB preventive effect of PPI in patients taking warfarin. Ray et al. [20], in large retrospective cohort study, found that PPI administration significantly reduced the risk of hospitalization for UGIB in patients receiving warfarin with concomitant antiplatelet drugs or NSAIDs (45% reduction (hazard ratio 0.55; 95% CI 0.39–0.77), *P* = 0.0004).

- In DAPT, we recommend the combined use of PPIs to prevent upper gastrointestinal bleeding (UGIB).
  Recommendation: strong, 100% agreed, evidence level A.
- If taking warfarin, we suggest using PPIs to prevent UGIB in patients taking antiplatelet drugs or NSAIDs in combination.
  Recommendation: weak, 100% agreed, evidence level C.
  (PPI is not covered by Japanese insurance for primary prevention of PUB in patients taking LDA or NSAIDs).
  Comment: MAs of RCTs aimed to determine whether the combination of PPIs is useful for the prevention of UGIB in DAPT with clopidogrel and aspirin. In addition to the large randomized COGENT trial [16], an MA was conducted involving three studies (two RCTs) [17, 18] comparing PPI usage. PPIs significantly prevented UGIB (risk ratio [RR] 0.26; 95% confidence interval [CI] 0.13–0.53, *P* = 0.0002) (Fig. 1). There was no significant difference in major adverse cardiovascular events, with or without PPI (RR 1.01; 95% CI 0.80–1.26, *P* = 0.96). In the 2017 focused update on DAPT in coronary artery disease of the European Society of Cardiology, PPIs in combination with DAPT were recommended (recommendation class I) [19].

## *H. pylori* eradication therapy

### First-line eradication therapy

#### CQ-5

What kind of regimen should we select for first-line *H. pylori* eradication therapy?

**Fig. 2:**
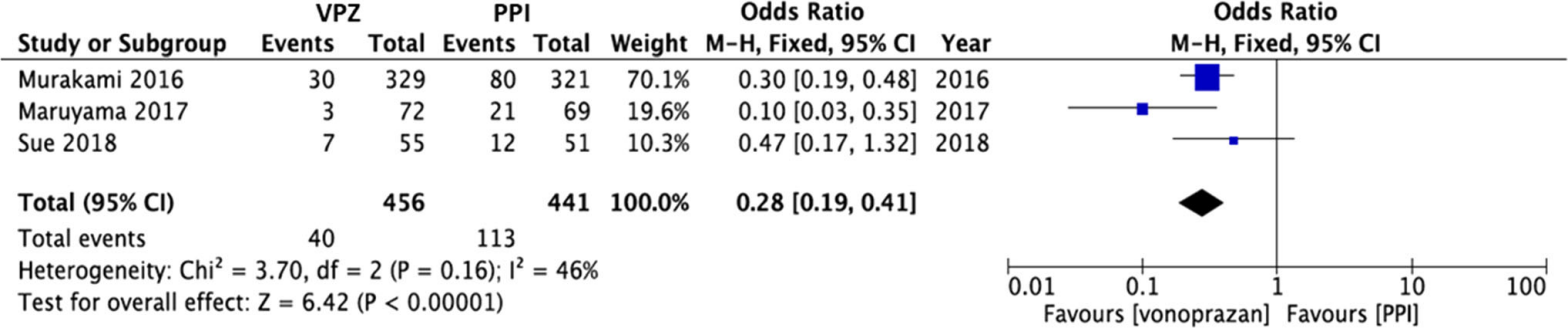
Forest plots of eradication rates of first-line therapy between vonoprazan-containing triple therapy and proton pump inhibitor (PPI)-containing triple therapy in the randomized control trials. In meta-analysis, the eradication rates of vonoprazan-containing triple therapy are significantly higher than that of PPI-containing triple therapy (odds ratio 0.28; 95% CI 0.19–0.41, *P* < 0.00001)

**Fig. 3:**
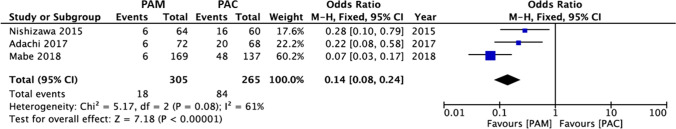
Forest plots of eradication rates of second-line therapy between proton pump inhibitor/amoxicillin/metronidazole (PAM) therapy and proton pump inhibitor/amoxicillin/clarithromycin (PAC) therapy in the randomized control trials in Japan. In meta-analysis, the eradication rates of PAM therapy are significantly higher than that of PAC therapy (odds ratio 0.14; 95% CI 0.08–0.24)

- As triple eradication therapy using VPZ with amoxicillin and clarithromycin has a high eradication rate compared with that of PPIs, VPZ is recommended as a first-line therapy.
  Recommendation: strong, 100% agreed, evidence level A.
- The recommended antibiotics for first-line therapy include amoxicillin, clarithromycin, or metronidazole. In Japan, the combination of amoxicillin and metronidazole is recommended due to the high incidence rate of clarithromycin-resistant strains (not covered by the Japanese insurance system).
  Recommendation: strong, 100% agreed, evidence level A.
- When PPIs are used, sequential therapy and concomitant quadruple therapy are suggested due to the high eradication rate in the first-line therapy compared with triple therapy (not covered by the Japanese insurance system).
  Recommendation: weak, 100% agreed, evidence level A.
  Comment: In the Japanese insurance system, triple therapy with a PPI or VPZ, amoxicillin, and clarithromycin is used as first-line therapy [21]. As the degree and duration of acid inhibition are related to the cure rate of *H. pylori* [22], an MA compared the efficacy of VPZ-containing therapies to PPI-containing therapies (Fig. 2) [23]. In areas with high clarithromycin-resistance rates (> 15%), the Maastricht V/Florence Consensus Report suggested that when culture/sensitivity testing is not performed before eradication, first-line therapy using clarithromycin should not be used; bismuth quadruple (PPI/bismuth/tetracycline/metronidazole) or concomitant quadruple therapy (PPI/amoxicillin/clarithromycin/nitroimidazole or metronidazole) is recommended [24]. In addition, in areas with low metronidazole- and high clarithromycin-resistance rates, PPI/amoxicillin/metronidazole has a higher eradication rate than PPI/amoxicillin/clarithromycin (Fig. 3) [25].

### Second-line eradication therapy

#### CQ-6

What regimen should we select for second-line *H. pylori* eradication therapy?

- Triple therapy with PPI/VPZ, amoxicillin, and metronidazole is recommended.
  Recommendation: strong, 100% agreed, evidence level A.
  Comment: Two MAs [26, 27] found that tetracycline/quinolone-based quintuple or quadruple therapies were more effective than other therapies. One RCT revealed that levofloxacin sequential therapy was more effective than levofloxacin triple therapy [28]. These drugs have not been approved for *H. pylori* eradication therapy in Japan. In addition, the rate of *H. pylori* infection with primary resistance to levofloxacin is high in Japan. The eradication rate of triple therapy with PPI/VPZ, amoxicillin, and metronidazole is still high [4], and does not differ between the two regimens [29–31].

### Third-line eradication therapy

#### CQ-7

What regimen should we select for third-line *H. pylori* eradication therapy?

- Triple therapy with PPI, sitafloxacin, and metronidazole or PPI, sitafloxacin, and amoxicillin is suggested (not covered by the Japanese insurance system).
  Recommendation: weak, 100% agreed, evidence level B.
  Comment: Three Japanese RCTs of third-line therapies showed that the eradication rates were 70.0–90.9% [32–34]. The eradication rate of triple therapy with PPI, sitafloxacin, and metronidazole was 70.0–88.9% [32–34], and that of PPI, sitafloxacin, and amoxicillin was 72.4–90.9% [33, 34]. Because these eradication rates were not high enough, these regimens are not recommended, but suggested.

### Ulcer recurrence after *H. pylori* eradication

#### CQ-8

Is maintenance treatment necessary for the recurrence of peptic ulcer after successful *H. pylori* eradication?

- When the cause of peptic ulcer recurrence is unclear, long-term maintenance treatment with PPIs or H_2_RAs is suggested.
  Recommendation: weak, 100% agreed, evidence level D.
  Comment: There are currently no published RCTs or MAs. Causes of the recurrence of peptic ulcers after successful *H. pylori* eradication include using LDA and NSAIDs, reinfection with *H. pylori*, and smoking habits. To prevent peptic ulcer recurrence, the exclusion of these factors is necessary. Idiopathic peptic ulcers are thought to be one of unknown causes of peptic ulcers after successful *H. pylori* eradication; therefore, long-term maintenance treatment with PPIs or H_2_RAs is suggested, when the cause of peptic ulcer recurrence is unclear.

## Non-eradication therapy

Initial therapy.

### Gastric ulcer

#### CQ-9

What is the first-line drug for the initial non-eradication treatment of gastric ulcers?

- Either PPIs or P-CAB is recommended.
  Recommendation: strong, 100% agreed, evidence level A.
- If PPIs and P-CAB cannot be prescribed, H_2_RAs are recommended.
  Recommendation: strong, 100% agreed, evidence level B.
- If PPIs and P-CAB cannot be prescribed, pirenzepine, sucralfate, and misoprostol are suggested.
  Recommendation: weak, 100% agreed, evidence level B.
- If the above drugs cannot be prescribed, gastric mucosa-protecting agents (excluding sucralfate and misoprostol) are suggested.
  Recommendation: weak, 100% agreed, evidence level B.
  Comment: We recommend either PPIs or P-CAB due to their demonstrated ulcer healing rate of PPIs being significantly higher than that of H_2_RAs [35–38]. P-CAB gained popularity in recent years due to their high ulcer healing rate for gastric ulcers compared with lansoprazole [39]. When PPIs and P-CAB cannot be prescribed, H_2_RAs are recommended. There were no reported significant differences in ulcer healing rates between H_2_RAs. Moreover, pirenzepine, sucralfate, and misoprostol are suggested as their ulcer healing rates are equivalent to those of H_2_RAs.

### Duodenal ulcer

#### CQ-10

What is the first-line drug for the initial non-eradication treatment of duodenal ulcer?

- Either PPIs or P-CAB is recommended.
  Recommendation: strong, 100% agreed, evidence level A.
- If PPIs and P-CAB cannot be prescribed, H_2_RAs are recommended.
  Recommendation: strong, 100% agreed, evidence level B.
- If PPIs and P-CAB cannot be prescribed, pirenzepine, sucralfate, and misoprostol are suggested.
  Recommendation: weak, 100% agreed, evidence level B.
  Comment: We recommend either PPIs or P-CAB as the ulcer healing rate of PPIs is significantly higher than that of H_2_RAs [40]. P-CAB gained popularity in recent years due to their high ulcer healing rate for duodenal ulcers compared with lansoprazole [39]. When PPIs cannot be prescribed, H_2_RAs are recommended.

## Drug-induced ulcer

Nonselective NSAID-induced ulcer.

### Treatment

#### CQ-11

How should NSAID-induced ulcers be treated?

**Fig. 4:**
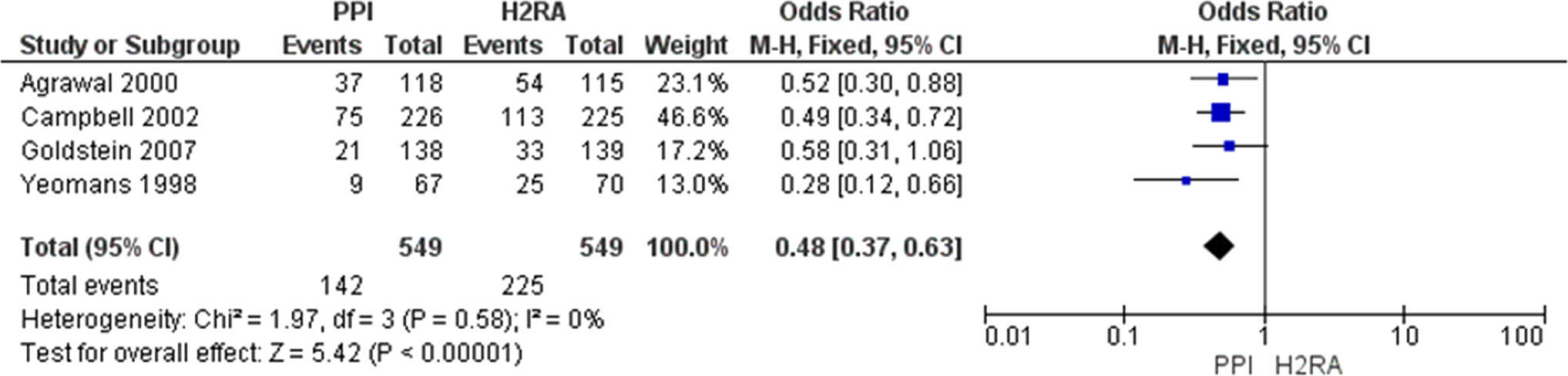
Meta-analysis of comparison of ulcer curative effect between PPI and H_2_RA under the NSAIDs continuation. In meta-analysis, the healing rate of peptic ulcers for 8 weeks is higher in the PPI groups than in the H_2_RA groups

- NSAIDs should be discontinued, and administration of anti-ulcer drugs is recommended.
  Recommendation: strong, 100% agreed, evidence level A.
- If NSAIDs cannot be discontinued, administration of PPIs is recommended as a first-line therapy.
  Recommendation: strong, 100% agreed, evidence level A.
  Comment: Gastric and duodenal ulcers in NSAID users heal to a high rate when NSAIDs are withdrawn [41]. In comparative studies of PPIs vs. H_2_RA [42] and PPIs vs. PG analogs [43], the healing rate of gastric and duodenal ulcers was highest in PPI groups. The original MA in this guideline indicated that the healing rate of peptic ulcers over 8 weeks was higher in PPI groups than in H_2_RA groups; thus PPIs are recommended as the first-line therapy (Fig. 4).

### Prevention

#### CQ-12

If a patient receiving NSAIDs tests positive for *H. pylori* infection, should *H. pylori* eradication therapy be administered?

- Eradication of *H. pylori* is recommended for prevention of ulcers in NSAID-naïve patients.
  Recommendation: strong, 100% agreed, evidence level A.
  Comment: In comparative studies of PPIs and *H. pylori* eradication, PPIs are superior to the eradication of *H. pylori* in preventing recurrent bleeding in patients taking NSAIDs [44].
  Studies have [45, 46] indicated that *H. pylori* eradication therapy reduces the incidence of ulcers in patients receiving NSAIDs; however, an effect of *H. pylori* eradication therapy on preventing peptic ulcer cannot be expected during NSAID therapy. In an MA reported in 2012, the efficacy of *H. pylori* eradication therapy for preventing peptic ulcer was observed, especially in naïve users and in Asian populations [46].

#### CQ-13

Is preventive therapy for NSAID-induced ulcers necessary in patients with no history of ulcers?

- Prevention of NSAID-induced ulcers by administration of PPIs is necessary and suggested even in patients with no history of ulcers.
  Recommendation: weak, 100% agreed, evidence level A.
  **(**PPIs are not covered by Japanese insurance for primary prevention of ulcers in patients taking NSAIDs).
  Comment: In patients receiving NSAID therapy for more than three months, the efficacy of PG analogs [47], PPIs [48], or high-dose H_2_RAs [49] given as primary preventions has been reported. Scally B et al. [50] showed that PPIs had larger protective effects than H_2_RAs or PG analogs for peptic ulcers and further bleeding; therefore, PPIs are suggested as a first-line therapy.

#### CQ-14

How should recurrence be prevented in patients with a history of ulcers or bleeding ulcers who are starting NSAID therapy?

**Fig. 5:**
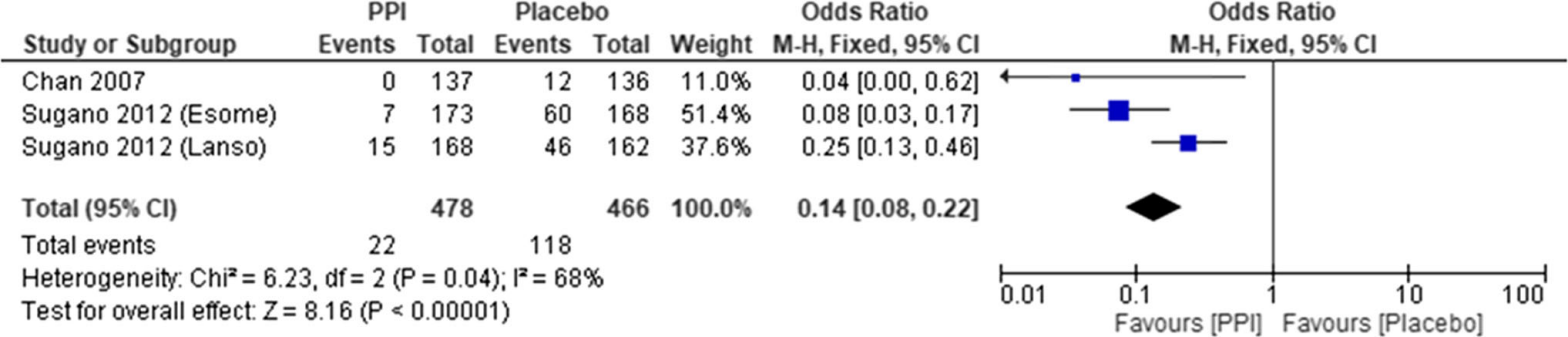
Meta-analysis of preventive effect in the secondary prevention of NSAIDs ulcer. In meta-analysis, the recurrence rate of peptic ulcers in patients with a history of gastric or duodenal ulcers who required long-term NSAID therapy is lower in the PPI groups than in the placebo groups

- PPIs are recommended, and VPZ is suggested to prevent NSAID-induced ulcers in patients with a history of ulcers.
  Recommendation: weak, 100% agreed, evidence level B.
- Concomitant administration of the selective cyclooxygenase (COX)-2 inhibitor with a PPI is recommended for preventing recurrence of bleeding NSAID-induced ulcers in patients with a history of bleeding ulcers.
  Recommendation: strong, 100% agreed, evidence level B.
  Comment: Although the efficacy of PG analogs [51] for secondary prevention was observed in high-risk patients with a history of peptic ulcers, patient drop-out due to diarrhea is common.
  Studies found that PPIs were superior to placebos in reducing the risk of gastric or duodenal ulcer recurrence in patients with a history of ulcers requiring long-term NSAID therapy [52, 53]. The original MA in this guideline indicated that the recurrence rate of peptic ulcers in patients with a history of ulcers requiring long-term NSAID therapy was lower in PPI groups than in placebo groups. Therefore, PPIs are recommended as the first-line therapy (Fig. 5).
  Chan et al. [54] suggested that combination treatment with a selective COX-2 inhibitor and a PPI was more effective than a selective COX-2 inhibitor alone for prevention of ulcer bleeding in patients at high risk.
  Mizokami et al. [6] reported that VPZ was not inferior to PPIs, indicating that VPZ can be recommended for the second prevention of NSAID-induced peptic ulcer as well as PPIs.

#### CQ-15

How should NSAID-induced ulcers be prevented in patients receiving high-dose NSAIDs or a combination of NSAIDs and antithrombotic drugs or glucocorticoids or bisphosphonates, who are elderly, or have severe complications?

- In patients receiving combinations of NSAIDs and glucocorticoids or antithrombotic drugs, administration of a COX-2 inhibitor is recommended for ulcer prevention.
  Recommendation: strong, 100% agreed, evidence level B.
- In elderly or patients with severe complications, administration of PPIs is recommended for the prevention of NSAID-induced ulcers.
  Recommendation: strong, 100% agreed, evidence level A.
  Comment: Concomitant use of nonselective NSAIDs or LDA, but not selective COX-2 inhibitors, with corticosteroids, or anticoagulants, increases the risk of UGIB, indicating that selective COX-2 inhibitors do not increase the risk of UGIB [55]. The efficacy of PPIs for prevention of complications has been demonstrated in a previous study [56].
  Selective NSAID (COX-2 selective inhibitor)-induced ulcers.

#### CQ-16

Is a COX-2 selective inhibitor useful for the prevention of NSAID-induced ulcers?

**Fig. 6:**
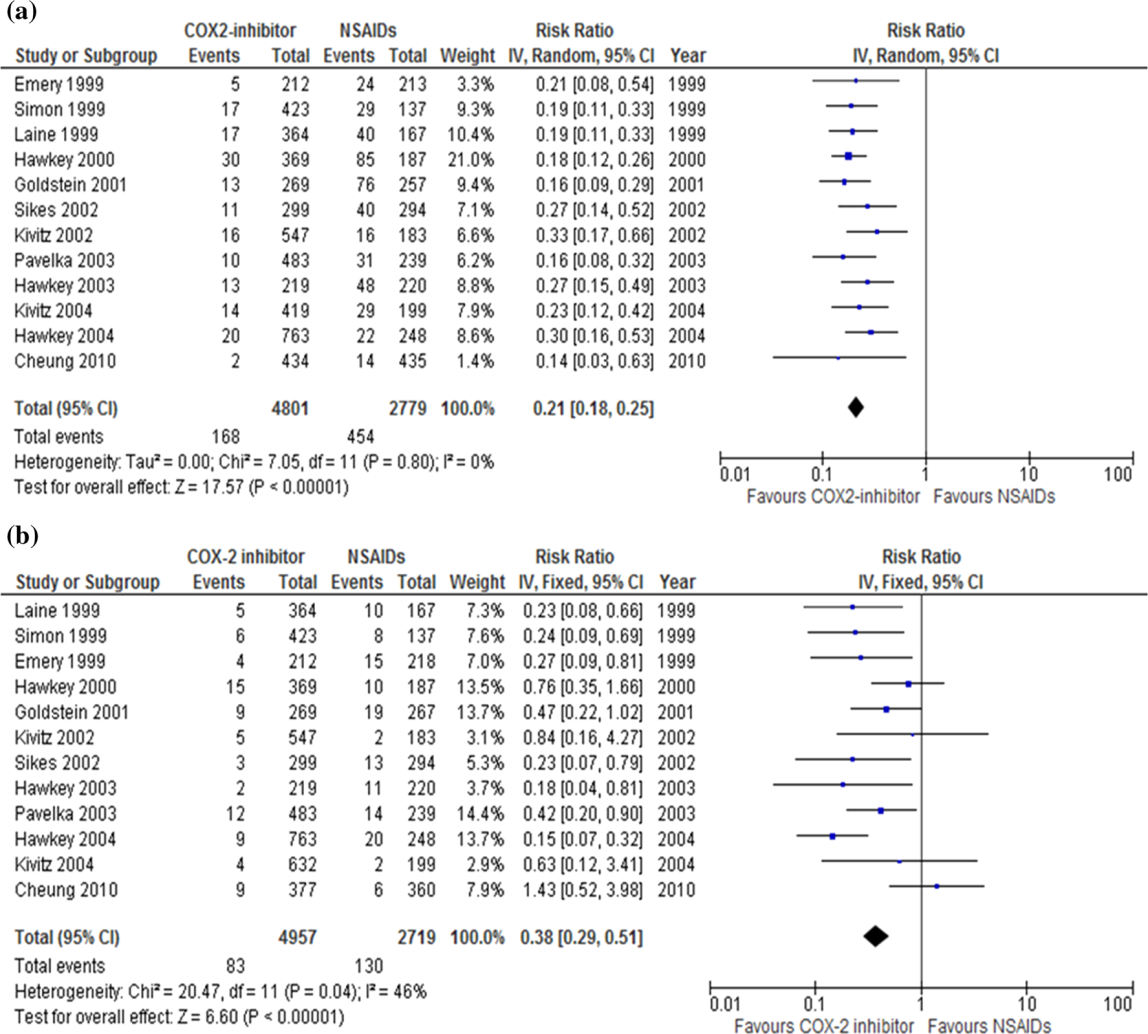
Forest plots of peptic ulcer risk between COX-2 selective inhibitor and NSAID therapy in the randomized control trials (**a** gastric ulcer, **b** duodenal ulcer). In meta-analysis, the incidence of gastric (risk ratio 0.21; 95% CI 0.18–0.25) (**a)** and duodenal ulcers (risk ratio 0.38; 95% CI 0.29–0.51) (**b**) are lower in patients using COX-2 selective inhibitors than in patients using NSAIDs

- COX-2 selective inhibitors are recommended for the prevention of NSAID-induced ulcers.
  Recommendation: strong, 100% agreed, evidence level A.
  Comment: The incidence rate of peptic ulcers in Western countries is significantly lower in patients taking COX-2 selective inhibitors than in patients taking nonselective NSAIDs [57–59]. We conducted an MA and found that the incidence of gastric ulcers (RR 0.21; 95% CI 0.18–0.25, *P* < 0.00001) (Fig. 6a) and duodenal ulcers (RR 0.38; 95% CI 0.29–0.51, *P* < 0.00001) (Fig. 6b) was lower in patients using COX-2 selective inhibitors than in patients using NSAIDs. Using COX-2 selective inhibitors had a lower risk of developing serious ulcer complications.
  A RCT including healthy Japanese volunteers showed that the incidence of gastroduodenal ulcers was 1.4%, 27.6%, and 2.7% in the celecoxib, loxoprofen, and placebo groups, respectively (*P* < 0.0001 in favor of the celecoxib group) [60]. We conducted an MA and found that the incidence of peptic ulcers was lower in patients using COX-2 selective inhibitors than in patients using NSAIDs (RR 0.13; 95% CI 0.04–0.44, *P* = 0.0010), similar to findings from patients in Western countries. These results indicate that COX-2 selective inhibitors are useful for the prevention of NSAID-induced ulcers and serious ulcer complications.

#### CQ-17

Is preventive medication with anti-ulcer agents required for patients taking a COX-2 selective inhibitor?

- Prevention with anti-ulcer agents is recommended for patients taking COX-2 selective inhibitors with a history of peptic ulcers or hemorrhage.
  Recommendation: strong, 100% agreed, evidence level B.
- No prevention with anti-ulcer agents is recommended for patients taking COX-2 selective inhibitors without a past history of peptic ulcer.
  Recommendation: strong, 100% agreed, evidence level B.
  Comment: An MA revealed that the incidence of drug-induced peptic ulcer development was similar between patients who received COX-2 selective inhibitors and placebos, suggesting that COX-2 selective inhibitors do not increase the risk of drug-induced peptic ulcers [60–62]. In a network MA, concomitant dosing of COX-2 selective inhibitors with PPIs effectively prevented the development of drug-induced peptic ulcer compared with COX-2 selective inhibitors alone [63]. However, patients with a history of peptic ulcers have a higher risk of developing drug-induced peptic ulcers.

## LDA-induced ulcer

### Treatment

#### CQ-18

How should LDA-related peptic ulcers be treated?

- Concomitant use of PPIs with continuous LDA therapy is recommended for LDA-related peptic ulcers.
  Recommendation: strong, 100% agreed, evidence level A.
  Comment: For patients with a history of LDA-related PUB, concomitant use of PPIs with continuous LDA therapy after endoscopic hemostasis was equivalent in the recurrence of PUB compared to concomitant use of placebos and PPIs. Furthermore, continuous LDA therapy reduces overall mortality rates related to cardiovascular (CV) events [8]. In the same study, concomitant use of PPIs with continuation of LDA therapy did not increase the incidence of PUB recurrence [8]. The peptic ulcer healing rate was similar between the PPI alone and the LDA combined with PPI [64].
  Prevention.

#### CQ-19

What kind of concomitant use of medicine should be effective for reducing the incidence and prevalence of LDA-related peptic ulcers?

**Fig. 7:**
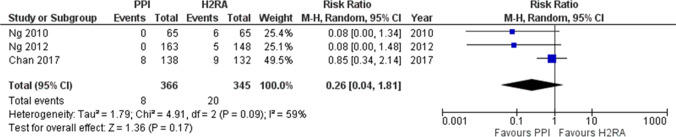
Effects of proton pump inhibitors (PPIs) and histamine 2-receptor antagonists (H_2_RAs) in preventing upper gastrointestinal (GI) ulcers related to low dose aspirin (LDA). An independently meta-analysis demonstrates that no significant difference (RR 0.26; 95% CI 0.04–1.81, *P* = 0.17) in the incidence of LDA-related upper GI ulcers between PPIs and H_2_RAs

- PPIs or H_2_RAs is recommended for the reduction of the incidence and prevalence of LDA-related peptic ulcers.
  Recommendation: strong, 100% agreed, evidence level A.
  **(**PPIs and H_2_RAs are not covered by Japanese insurance for primary prevention of ulcers in patients taking LDA).
  (H_2_RAs are not covered by Japanese insurance for secondary prevention of ulcers in patients taking LDA).
  Comment: MA indicated that LDA treatment with concomitant use of H_2_RAs or PPIs reduced the risk of LDA-related peptic ulcers [65, 66]. PPIs were superior to H_2_RAs in preventing upper GI ulcers related to LDA [67]. However, a RCT by Chan et al. [68] indicated that there were no significant differences between rabeprazole and famotidine in the incidence of recurrent ulcers in patients taking LDA. We used data from two RCTs [69, 70] in Mo et al. [67] and one RCT [68] to independently conduct an MA. We found no significant difference (RR 0.26; 95% CI 0.04–1.81, *P* = 0.17) in the incidence of LDA-related upper GI ulcers between PPIs and H_2_RAs (Fig. 7).

#### CQ-20

What kind of concomitant use of medicine should be effective for reducing the incidence and prevalence rate of LDA-related PUB?

**Fig. 8:**
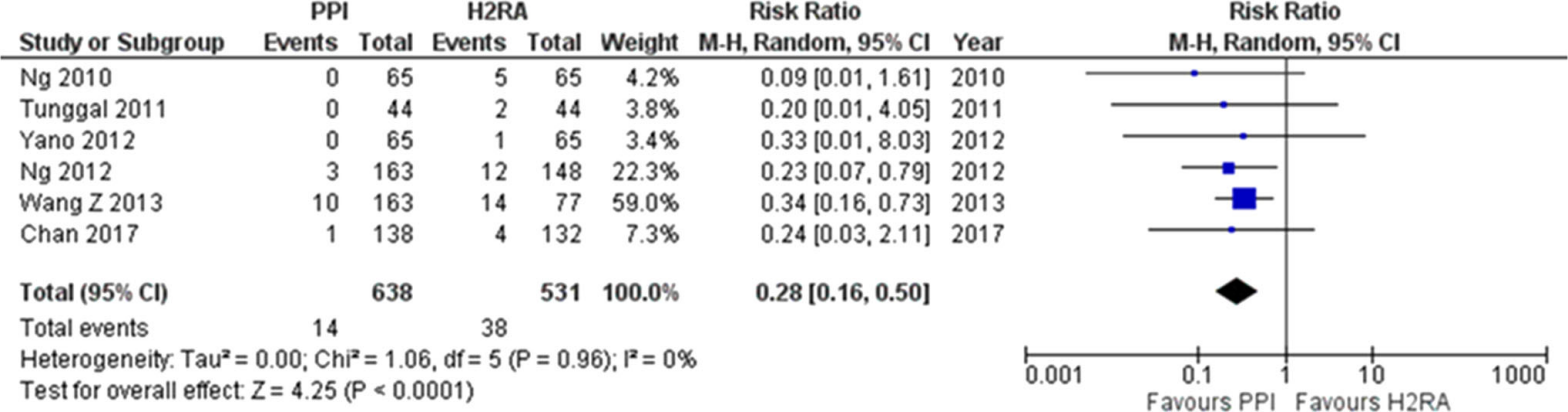
Effects of proton pump inhibitors (PPIs) and histamine 2-receptor antagonists (H_2_RAs) in preventing upper gastrointestinal (GI) ulcer bleeding related to low dose aspirin (LDA). An independently meta-analysis demonstrated that PPIs are superior to H_2_Ras (RR 0.28; 95% CI 0.16–0.50, *P* < 0.0001) in prevention of LDA-related upper GI bleeding

- PPIs or VPZ is recommended for the reduction of the incidence and prevalence of LDA-related PUB.
  Recommendation: strong, 100% agreed, evidence level A.
  **(**PPI and VPZ are not covered by Japanese insurance for primary and secondary prevention of PUB in patients taking LDA).
  Comment: MA revealed that LDA treatment with concomitant use of H_2_RAs or PPIs reduced the risk of LDA-related PUB [65, 71]. PPIs were superior to H_2_RAs in preventing upper GI ulcer bleeding related to LDA [66, 67]. We used data from three RCTs [68–70] in Mo et al. [67] and three RCTs [72–74], which compared PPIs and H_2_RAs to independently conduct an MA. We found that PPIs were superior to H_2_RAs (RR 0.28; 95% CI 0.16–0.50, *P* < 0.0001) in the prevention of LDA-related upper GI bleeding (Fig. 8). Furthermore, the incidence of bleeding in the stomach or duodenum was significantly lower with both 10 mg and 20 mg of VPZ compared to 15 mg of lansoprazole in patients taking LDA with a history of ulcers [7].

#### CQ-21

What kind of concomitant use of medicine should be effective for reducing the incidence and prevalence rate of recurrent LDA-related PUB?

- PPIs in addition to the eradication of *H. pylori* infection are recommended for the reduction of the incidence and prevalence rate of LDA-related PUB.
  Recommendation: strong, 100% agreed, evidence level B.
  **(**PPI is not covered by Japanese insurance for second prevention of peptic ulcer bleeding in patients taking LDA).
- H_2_RAs in addition to the eradication of *H. pylori* infection are suggested for the reduction of the incidence and prevalence rate of LDA-related PUB.
  Recommendation: weak, 100% agreed, evidence level C.
  (H_2_RAs are not covered by Japanese insurance for second prevention of PUB in patients taking LDA).
  Comment: The eradication of *H. pylori* is equivalent in the probability of recurrent bleeding to treatment with omeprazole in patients receiving LDA [75]. In patients with ulcer complications related to the long-term use of LDA, treatment with lansoprazole in addition to the eradication of *H. pylori* significantly reduced recurrence rates of ulcer bleeding compared to placebo plus *H. pylori* eradication [76]. In patients with LDA-related peptic ulcers, high-dose famotidine therapy is inferior to pantoprazole in preventing recurrent ulcer bleeding that continues to receive LDA [69]. LDA treatment with concomitant use of rabeprazole showed no difference in the incidence of recurrent upper GI ulcer bleeding rates compared to famotidine in patients with a history of LDA-related peptic ulcer bleeding [68].

#### CQ-22

How should LDA-related peptic ulcer recurrence be prevented in patients with a history of peptic ulcers?

- PPI or VPZ is recommended to reduce the recurrence rate of LDA-related peptic ulcers.
  Recommendation: strong, 100% agreed, evidence level A.
- H_2_RAs are suggested to reduce the recurrence rate of LDA-related peptic ulcers.
  Recommendation: weak, 100% agreed, evidence level C.
  Comment: In patients with a history of LDA-related peptic ulcers who continued to receive LDA, pantoprazole was superior to famotidine in preventing recurrent bleeding ulcers [69]. LDA treatment with concomitant use of 20 mg rabeprazole showed no difference in the recurrence of upper GI ulcer bleeding compared to 40 mg of famotidine in patients with a history of LDA-related PUB [68]. An independent MA of four RCT articles [77–80] demonstrated that PPIs were superior to control drugs, placebos, gefarnate, and teprenone, in preventing the recurrence of LDA-related upper GI ulcers (RR 0.09; 95% CI 0.06–0.15, *P* < 0.00001). A study found that 47–53% of patients with gastroduodenal ulcer scars on endoscopy at baseline, famotidine was superior to placebo in prevention of the incidence of gastroduodenal ulcers in patients taking LDA [81]. An RCT by Kawai et al. indicated that 10 mg of VPZ was superior to 15 mg of lansoprazole in the prevention of peptic ulcer recurrence in patients with a history of peptic ulcers who required LDA [7].

#### CQ-23

In patients without a history of peptic ulcer, is the prevention of LDA-related peptic ulcers necessary?

- PPIs are recommended for the primary prevention of LDA-related peptic ulcers without a history of ulcers.
  Recommendation: strong, 82% agreed, evidence level A.
  Comment: In an RCT by Takeuchi et al., famotidine was superior to teprenone in reducing the number of erosions during LDA use in 94% of patients without a history of peptic ulcers [82]. Scheiman et al. [83] indicated that esomeprazole reduced the occurrence of peptic ulcers in patients taking LDA in 73% of patients without a history of peptic ulcers. Ng et al. [70] found that esomeprazole was superior to famotidine in preventing upper GI complications related to LDA, clopidogrel, and thrombolytics in 95% of patients without a peptic ulcer.

#### CQ-24

Can COX-2 selective inhibitors reduce the risk of peptic ulcers compared to nonselective NSAIDs when administered with LDA?

- COX-2 selective inhibitors reduce the risk of peptic ulcers and bleeding in patients taking LDA compared to nonselective NSAIDs.
  Recommendation: strong, 100% agreed, evidence level A.
- Concomitant use of celecoxib with PPIs is recommended for the prevention of gastric injury in patients with moderate or lower risk of peptic ulcers requiring LDA and NSAIDs.
  Recommendation: strong, 100% agreed, evidence level A.
  Comment: Concomitant use of a COX-2 selective inhibitor with LDA increased the incidence of peptic ulcers [84], whereas COX-2 selective inhibitors lowered the risk of peptic ulcers compared to nonselective NSAIDs when using LDA [85, 86]. In patients taking LDA, the use of celecoxib or naproxen with PPIs resulted in similar rates of gastroduodenal ulceration [87]. In patients at high risk of both CV and GI events requiring concomitant LDA and NSAIDs, celecoxib with PPIs was the preferred treatment to reduce the risk of recurrent upper GI bleeding compared to naproxen with esomeprazole [88]. A study found that in 45% of arthritis patients taking LDA, the risk of GI events was lower with celecoxib than with naproxen or ibuprofen [89]. COX-2 selective inhibitors and nonselective NSAIDs increased the risk and incidence of CV events [90], whereas celecoxib was non-inferior to naproxen or ibuprofen with regard to CV safety [88, 89]. In addition, the American College of Gastroenterology (ACG) guidelines suggest that concomitant use of COX-2 selective inhibitors or nonselective NSAIDs in patients taking LDA should not be prescribed in patients at high risk of peptic ulcer [91].

#### CQ-25

Is PPI recommended for the prevention of recurrence of peptic ulcers with NSAID treatment in patients taking LDA?

- Celecoxib with concomitant use of PPI is recommended for the prevention of peptic ulcer recurrence after NSAID treatment in patients taking LDA.
  Recommendation: strong, 100% agreed, evidence level A.
  **(**PPI is not covered by Japanese insurance for the primary prevention of ulcers in patients taking LDA).
  Comment: In patients with a history of ulcer who are receiving combinations of NSAIDs and LDA, concomitant use of PPI lowered the risk for gastric ulcer recurrence as misoprostol [92]. In addition, Goldstein et al. indicated that naproxen with esomeprazole lowered the incidence of gastric ulcers compared to naproxen in LDA users [93]. There were fewer GI events in patients using both COX-2 inhibitors and LDA than nonselective NSAIDs and LDA [85, 86], and there were no differences in the occurrence of CV events between celecoxib and naproxen or ibuprofen [88, 89].

### Non-*H. pylori*, non-NSAIDs ulcer

#### CQ-26

How should non-*H. pylori* and non-NSAIDs ulcers be treated?

- PPIs are suggested for the initial treatment of non-*H. pylori* and non-NSAID idiopathic ulcers with PPIs or H_2_RAs for the prevention of recurrence.
  Recommendation: weak, 100% agreed, evidence level: C.
  Comment: As hyperacidity and hypergastrinemia have been observed in patients with idiopathic ulcers [94], we suggest PPIs as the initial treatment. However, Kanno et al. [95] reported that the healing rate at 12 weeks with PPIs was 77.4% for idiopathic ulcers compared to 95.0% for *H. pylori* ulcers. Therefore, PPIs may be insufficient for the treatment of idiopathic ulcers. Wong et al. [96] reported that the cumulative recurrence rate of the idiopathic ulcer group for 7 years without preventive treatment was 42.3%, higher than that of the *H. pylori* ulcer group (11.2%). Furthermore, in the following RCT conducted by them, they compared the efficacy of PPI and H_2_RA in preventing recurrence of idiopathic ulcers and reported that the cumulative incidence of upper GI bleeding at 24 months was 0.88% for PPI (lansoprazole: 30 mg once per day) and 2.63% for H_2_RA (famotidine: 40 mg once per day) The effect was not significantly different between the two groups (*P* = 0.336) [97]. Therefore, we suggest that both PPIs and H_2_RAs are candidates for the prevention of recurrence.

### Remnant gastric ulcer

#### CQ-27

What is the treatment for ulcers in the gastric remnant?

- PPI treatment is recommended for ulcers in the gastric remnant.
  Recommendation: strong, 100% agreed, evidence level C.
  Comment: The first choice for the treatment of ulcers in gastric remnant is drug therapy. In an open-label trial comparing omeprazole, cimetidine, sucralfate, colloidal bismuth, and misoprostol for ulcers in the gastric remnant, omeprazole was the best in terms of cure rate and cure speed [98]. The cure rates of ulcer in 2 weeks’ treatment were 66.7, 43.3, 22.2, 22.2, and 16.7% for omeprazole, cimetidine, sucralfate, colloidal bismuth, and misoprostol, respectively.
  To date, there has not been a RCT regarding the *H. pylori* eradication effect. However, in several cross-sectional studies comparing the *H. pylori* positivity rate between gastric remnants with and without ulcers, there was no difference [99–102]. From these results, the eradication effect for ulcer occurrence in the gastric remnant is unknown. The preventive effect of eradication for carcinogenesis in the gastric remnant is also undefined. However, speculating from histological improvement after eradication, it would be effective for preventing carcinogenesis. We should be cautious about the recovery of acid secretion after eradication for ulcer occurrence in the gastric remnant.

### Surgical treatment

#### CQ-28

Is eradication of *H. pylori* recommended after surgery for peptic ulcers?

- Eradication of *H. pylori* is recommended after omental patch or omental filling procedure for peptic ulcers if *H. pylori* positive.
  Recommendation: strong, 100% agreed, evidence level A.
  Comment: For post-surgery treatment, reports regarding eradication therapy were found, but not for PPI or H_2_RA treatment. Several reports were in agreement with the recommendation of eradication therapy for prevention of peptic ulcer recurrence after stomach preserving surgery [103–107]. El-Nakeeb et al. [107] indicated that early eradication after omental patch surgery for duodenal ulcer perforation is recommended for accelerating ulcer healing 8 weeks after surgery.
  Preventive effects for ulcer recurrence by eradication after gastrectomy were not consistent between reports. Further analysis is needed to determine the effect of acid secretion recovery after eradication of peptic ulcers.

#### FRQ-1

What is the treatment for an ischemic duodenal ulcer?

- Suggested PPIs or misoprostol as conservative treatments. Background conditions such as thrombosis and arterial stenosis should be investigated. IVR or surgery may be considered if the patient’s condition is exacerbated following conservative treatment.
  Comment: Fourteen case reports regarding ischemic duodenal ulcers were identified. Symptoms included acute onset abdominal pain, melena or hematemesis, chronic abdominal pain, and weight loss. Typical endoscopic findings for ischemia were longitudinal ulcers, and edematous and reddish changes in the surrounding mucosa other than the ulcer. The causes of acute ischemia include embolization for GI bleeding hemostasis or embolization therapy for hepatocellular carcinoma, systemic thromboembolism [108, 109], and unknown cases. Chronic symptoms were reported in patients at risk of atherosclerotic disease and severe stenosis of the celiac artery/superior mesenteric artery. Fasting and acid-secreting inhibitors have been used as conservative treatments. However, the clinical effectiveness of acid suppression in the posterior part of the bulb, which is exposed to bile and pancreatic juice, remains unclear. From the viewpoint of mucosal protection, misoprostol is suggested. In the case of acute onset ischemic ulcers, careful observation is required, considering the possibility of systemic thrombosis.

## Therapeutic algorithm

**Fig. 9:**
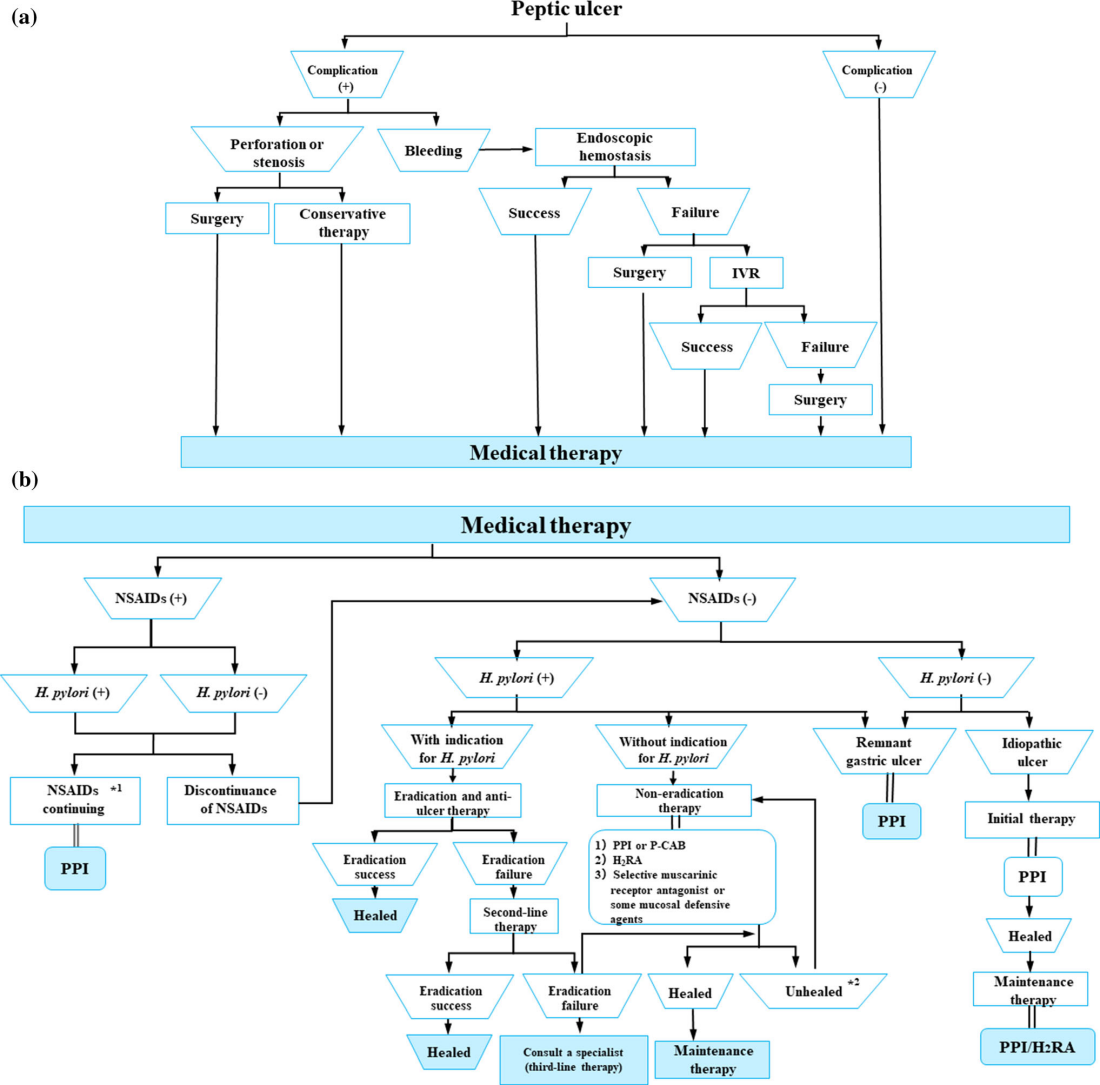
Algorithm for the treatment of peptic ulcer disease

Figure 9 shows the algorithm for the treatment of peptic ulcer disease. If complications are present, they are addressed prior to the treatment of the ulcer. Perforation or stenosis is treated with surgery or conservative therapy. Hemorrhagic ulcers are treated via endoscopic hemostasis. When endoscopic hemostasis failed, surgery or IVR is performed. When no complications are present, medical therapy is provided immediately (Fig. 9a).

NSAIDs are discontinued, and anti-ulcer therapy is provided for the treatment of NSAID-induced ulcers. If NSAIDs cannot be discontinued, the ulcer should be treated with a PPI. Patients with peptic ulcers who do not use NSAIDs should be tested for *H. pylori*. Eradication therapy is recommended for patients who are *H. pylori* positive. If first-line therapy fails, second-line therapy is provided. If the second-line therapy fails, a specialist is consulted for third-line therapy. For patients without an indication for eradication therapy, non-eradication therapy is provided followed by maintenance therapy to prevent ulcer recurrence. A remnant gastric ulcer is treated with a PPI, and an idiopathic ulcer is treated with PPI followed by maintenance therapy with a PPI or H_2_RA (Fig. 9b).

*^1^Contraindications. Only when administration is unavoidable as it cannot be discontinued.

*^2^Patients who do not use NSAIDs and are *H. pylori* negative are considered to have idiopathic peptic ulcers.

**Fig. 10:**
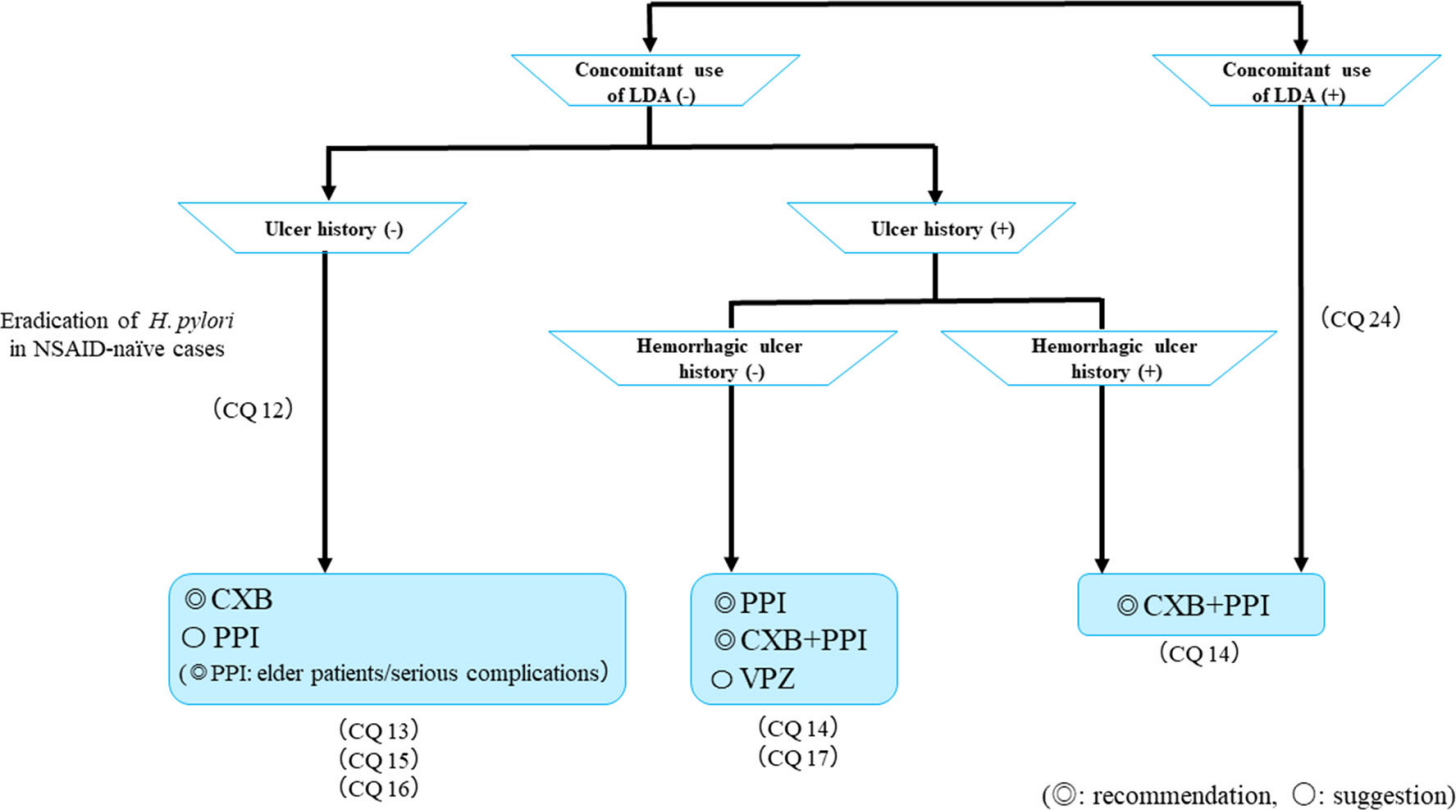
Algorithm for the prevention of NSAID-induced ulcers

Figure 10 shows the algorithm for the prevention of NSAID-induced ulcers. In patients with no ulcer history receiving NSAID therapy, celecoxib (CXB) is recommended and PPIs are suggested for ulcer prevention. However, PPIs are recommended for elderly patients and patients with serious complications. If patients are both NSAID-naïve and *H. pylori* positive, eradication therapy is recommended. For patients with a history of ulcers that do not include bleeding who are receiving NSAID therapy, a PPI with or without CXB is recommended, and the administration of VPZ is suggested for the prevention of ulcer recurrence. For patients with a history of hemorrhagic ulcers who are receiving NSAID therapy, CXB with a PPI is recommended. CXB with a PPI is recommended for patients receiving combined NSAID therapy and LDA.

**Fig. 11:**
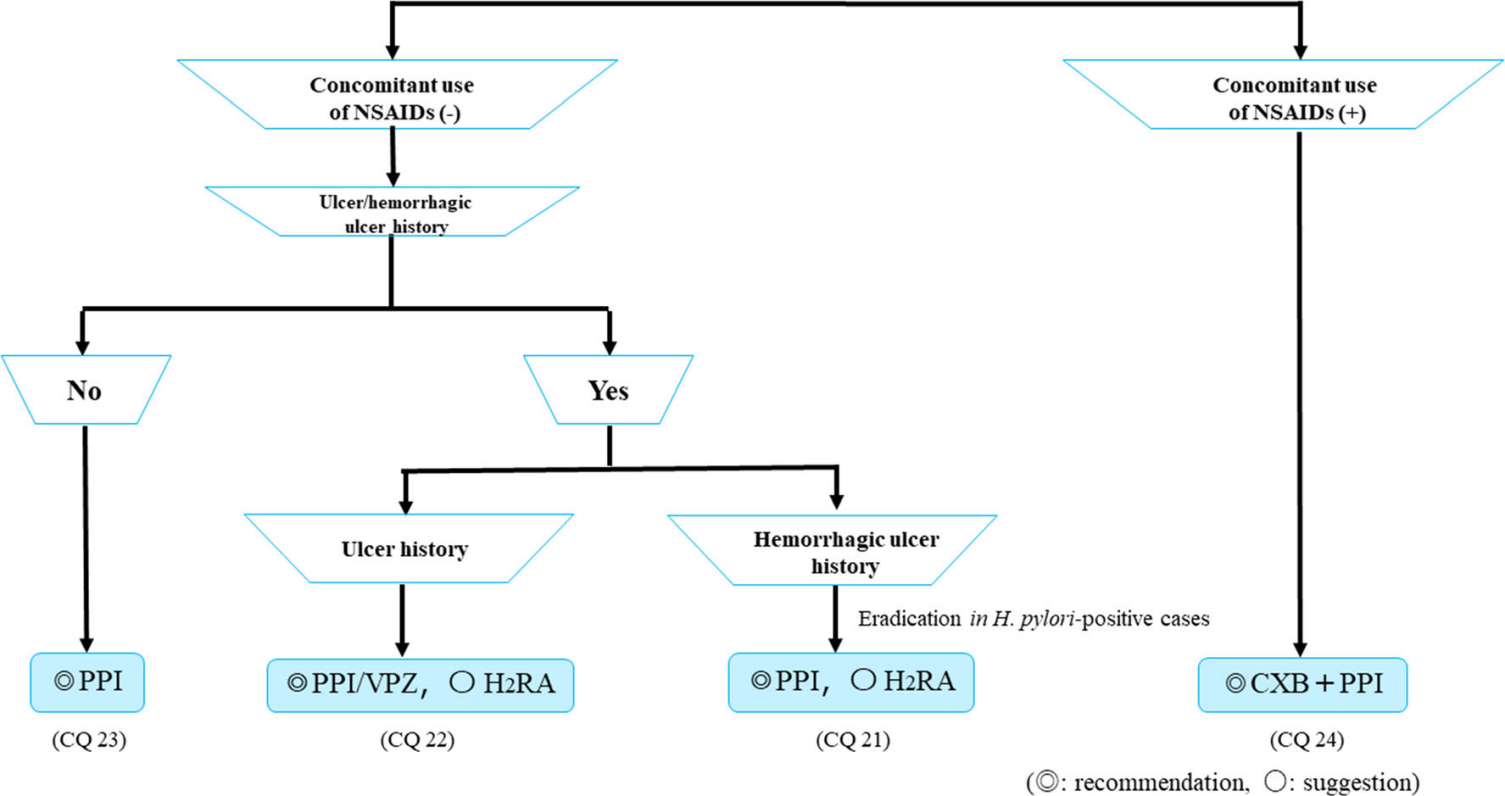
Algorithm for the prevention of LDA-related ulcers

Figure 11 shows the algorithm for the prevention of LDA-related ulcers. PPIs are recommended for ulcer prevention in patients receiving LDA therapy with no ulcer history. For patients with a history of ulcers without bleeding who are receiving LDA therapy, PPIs or VPZ are recommended and H_2_RAs are suggested. A PPI is recommended and H_2_RA is suggested for patients with a history of hemorrhagic ulcers who are receiving LDA therapy. However, if the patients are *H. pylori* positive, eradication therapy is recommended. Finally, for patients receiving combinations of LDA and NSAID therapy, CXB with a PPI is recommended.

## Appendix

Members of the Guidelines Committee who created and evaluated the JSGE ‘‘Evidence-based clinical practice guidelines for peptic ulcer disease 2020′’ are listed below.

## Executive committee

Chair: Kiichi Satoh (Department of Gastroenterology, International University of Health and Welfare Hospital). Vice-Chair: Tomoari Kamada (Department of Health Care Medicine, Kawasaki Medical School). Members: Toshiyuki Itoh (Education Center for Medicine and Nursing, Shiga University of Medical Science), Masanori Ito (Department of General Internal Medicine, Hiroshima University Hospital), Junichi Iwamoto (Department of Gastroenterology, Tokyo Medical University Ibaraki Medical Center), Tadayoshi Okimoto (Department of Gastroenterology, Faculty of Medicine, Oita University), Takeshi Kanno (Division of Gastroenterology, Tohoku University Hospital), Mitsushige Sugimoto (Department of Gastroenterological Endoscopy, Tokyo Medical University Hospital), Toshimi Chiba (Division of Internal Medicine, Department of Oral Medicine, Iwate, Medical University), Sachiyo Nomura (Department of Gastrointestinal Surgery, Graduate School of Medicine, The University of Tokyo), and Mitsuyo Mieda (Division of Gastroenterology, Department of Medicine, Jichi Medical University).

## Evaluation committee

Chair: Hideyuki Hiraishi (Niigataseirou Hospital).

Vice-Chair: Junji Yoshino (Fujita Medical University).

Member: Atsushi Takagi (Kameda Morinosato Hospital).

## The Japanese Society of Gastroenterology

President: Kazuhiko Koike (Department of Gastroenterology, Graduate School of Medicine, The University of Tokyo). Past President: Tooru Shimosegawa (South Miyagi Medical Center). Director Responsible: Hiroto Miwa (Division of Gastroenterology, Department of Internal Medicine, Hyogo College of Medicine), Nobuyuki Enomoto (First Department of Internal Medicine, Faculty of Medicine, University of Yamanashi).

## SR collaborator

Ryo Ogawa (Department of Gastroenterology, Faculty of Medicine, Oita University), Sotaro Ozaka (Department of Gastroenterology, Faculty of Medicine, Oita University), Yoshinari Kawahara (Department of Gastroenterology, Faculty of Medicine, Oita University), Xiaoyi Jin (Division of Gastroenterology, Tohoku University Hospital), Naoki Sumi (Department of Health Care Medicine, Kawasaki Medical School), Kenichiro Nakagawa (Division of Gastroenterology, Tohoku University Hospital), Kensuke Fukuda (Department of Gastroenterology, Faculty of Medicine, Oita University).

Tomoyuki Boda (Department of Gastroenterology and Metabolism, Graduate School of Biomedical and Health Sciences, Hiroshima University), Hitomi Mizuno (Toyoda Aoba Clinic), Masaki Murata (Department of Gastroenterology, National Hospital Organization, Kyoto Medical Center), Mohammad Yaghoobi (Division of Gastroenterology, Health Sciences Centre, McMaster University, Canada), and Yuan Yuhong (Division of Gastroenterology, Health Sciences Centre, McMaster University, Canada).

## Cooperative society

The Japanese Gastroenterological Association.

Japan Gastroenterological Endoscopy Society.
